# Theoretical analysis of expanded porphyrins: Aromaticity, stability, and optoelectronic properties

**DOI:** 10.3389/fchem.2022.948632

**Published:** 2022-09-01

**Authors:** Wei Wei, Zeng-Xia Zhao, Bao-Hui Xia, Wei Li

**Affiliations:** ^1^ Institute of Theoretical Chemistry, College of Chemistry, Jilin University, Changchun, China; ^2^ College of Chemistry, Jilin University, Changchun, China; ^3^ School of Chemistry and Materials Science, Hunan Agricultural University, Changsha, China

**Keywords:** DFT, aromaticity, stability, optoelectronic properties, expanded porphyrins

## Abstract

Expanded porphyrin systems are capable of binding a variety of substrates due to their increased cavity size and aromatic nature, holding important applications as magnetic resonance imaging contrast agents and as sensitizers for photodynamic therapy. It is there of fundamental interest to know the photoelectrical properties of expanded porphyrins using quantum chemistry calculations. In this work, we theoretically designed and screened a series of expanded porphyrins by incorporating terthiophene (TTH) and dithienothiophene (DTT) moieties. Our calculations showed that all the designed molecules exhibit excellent optoelectronic performance than the reference molecule. It is suggested that the porphyrin molecule with TTH moiety has better stability than the one with DTT moiety. Finally, we demonstrated that molecule **2** features with TTH moiety and the inverted selenophene ring outperform other molecules because it exhibits increased HOMO-LUMO gap, planar geometry, and strengthened aromaticity. We expect that this work can provide theoretical guidelines for the design of novel porphyrin materials.

## Introduction

Aromaticity is a fundamental and important concept in the field of chemistry with wide applications (Shen et al., 2018). Many studies have been focusing on the aromatic molecule, benzene (Gil-Guerrero et al., 2018). Aromaticity is directly related to the stability of molecules. In general, aromatic compounds have high thermodynamic stability and are substantially more stable than antiaromatic compounds. Although aromaticity cannot be directly measured experimentally, various parameters based on geometric (Listunov et al., 2018), thermodynamics (Nowroozi1 and Rad, 2017; Rakhi and Suresh, 2016), magnetic (Najmidin et al., 2013; Torrent-Sucarrat et al., 2017), and electronic properties can be used to evaluate aromaticity and antiaromaticity of molecules. In particular, the nucleus-independent chemical shift (NICS) analysis is the most popular method for determining the aromaticity of molecules due to its simplicity and effectivity (Schleyer et al., 1996). In addition to NICS, the highest occupied molecular orbital (HOMO)-lowest unoccupied molecular orbital (LUMO) gap value also has been used to characterize the aromaticity of a molecule (Bédé et al., 2018).

Porphyrin is a good aromatic compound (Cao et al., 2014; Cyrański et al., 2003; Wei et al., 2019; Wei et al., 2020). Porphyrins with 18 π-electrons have been under intense investigation due to their enormous potential for applications in catalysis, magnetic resonance imaging, and photodynamic therapy (Drain et al., 2009; Bryden and Boyle, 2016; Barona-Castaño et al., 2016; Tang et al., 2019). Porphyrin frameworks are composed of four pyrrole subunits that are connected in a coplanar fashion at their α-carbon atoms *via* a methine carbon bridge. Expanded porphyrins exhibit structures, electronic properties, coordination chemistry, and reactivities that are entirely different from those of porphyrins. Expanded porphyrins exhibit intriguing Hückel and Mobius topologies than tetrapyrrolic porphyrin macrocycles in view of their larger-sized macrocyclic core (Alka et al., 2019), presenting ideal candidates for novel nonlinear optical (NLO) switching devices (Desmedt et al., 2021). Since the first report by Johnson et al., sapphyrins have been well investigated in therapeutic potential due to their optical and material properties (Chatterjee et al., 2017). Compared to porphyrin, sapphyrin contains an additional pyrrole inserted between a meso-carbon and an α-pyrrolic position. There are also modified sapphyrins, that is, oxasapphyrins and dithiabenzisapphyrin (Richter and Lash, 1998; Panda et al., 2005; Jeong et al., 2008), with pyrrolic subunits replaced by other building fragments, exhibiting unusual aromaticity, stability, and absorption properties (Karthik et al., 2014; Szyszko et al., 2017; Rao et al., 2019).

Terthiophene (TTH) has been widely used as the π-conjugated building fragments to substitute pyrrole unit in porphyrin skeleton (Moriaty et al., 1985). Dithienothiophene (DTT), which contains two thiophene rings fused by a bridging sulfur atom, is an electron-rich moiety with the rigid fragment that has been used in many optoelectronic materials (Frey et al., 2002). Both TTH and DTT have three thiophene rings, and the difference is that TTH has the C-C single bond bridge. Therefore, the incorporation of TTH and DTT subunits is a promising way to improve the optoelectronic characteristics of porphyrin molecules. The X-ray analysis of sapphyrins structure reveals that the larger core size and the presence of lighter heteroatoms (i.e., N or O) adjacent to the heterocyclic rleadeads to an inverted structure, while the presence of heavier heteroatoms (i.e., S or Se) leads to the conventional structure (Misra and Chandrashekar, 2008). It would be interesting to investigate how this strategy will change the aromaticity, stability, and optoelectronic properties of sapphyrins.

**SCHEME 1:**
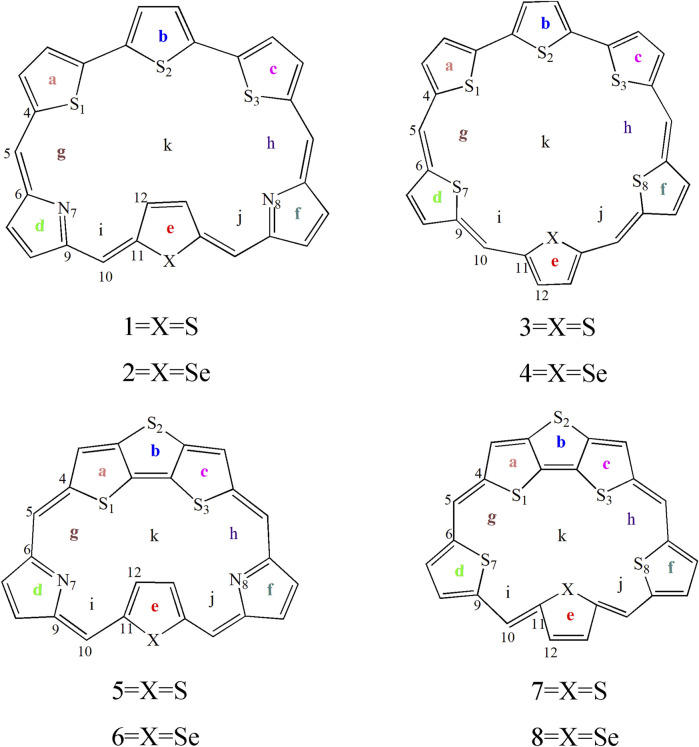
Sketch structures of molecules of **1**–**8**.

In this work, we theoretically designed eight macrocycle molecules by incorporating TTH and DTT subunits, coded as **1**–**8**, Scheme 1. The aromaticity, stability, and photophysical properties of the designed molecules are investigated by using density functional theory (DFT) and time-dependent DFT (TD-DFT) calculations. We also investigated the substitutions of the pyrrole N atom by heavier S and Se atoms in expanded porphyrins. This study aims to elucidate the impact of structural modifications on the overall performance of these expanded porphyrins. We expect the current theoretical work can pave the way for the design of novel porphyrin-based optoelectronic materials.

## Computational details

All calculations are performed with DFT/TD-DFT methods, as implemented in the Gaussian 09 software (Frisch et al., 2009). Geometry optimization was carried out using the DFT-B3LYP/6-311G (d,p) level of theory. Vibrational frequencies are calculated for the optimized structure at the same theory level to confirm that there are no imaginary frequencies and that the obtained structure is in local energy minima. Vertical electronic excitations are calculated using TD-DFT/CAM-B3LYP functional together with a 6-311G (d,p) basis set. Based on the optimized ground state structures, the NICS (1) values are calculated by means of the “atoms in molecules” theory, as implemented in the AIM2000 package (Bader et al., 1994). In this work, a few functionals (CAM-B3LYP, M05-2X, M06-2X) are performed on the calculation. The NICS (1) values with CAM-B3LYP are very close to M05-2X and M06-2X functionals. Therefore, CAM-B3LYP functional is the best choice due to its commonly used. Aromatic stabilization energy (ASE) is also used to evaluate the stability and aromaticity of molecules. ASE is an energy-based criterion for analyzing aromaticity. The ASE values can be calculated according to a ring-opening isobond chemical reaction, as we will introduce below. The molecule is aromatic if the reaction energy is positive, otherwise, the molecule is antiaromatic. Readers can refer to ref. 29 for more details.

## Results and discussion

**FIGURE 1:**
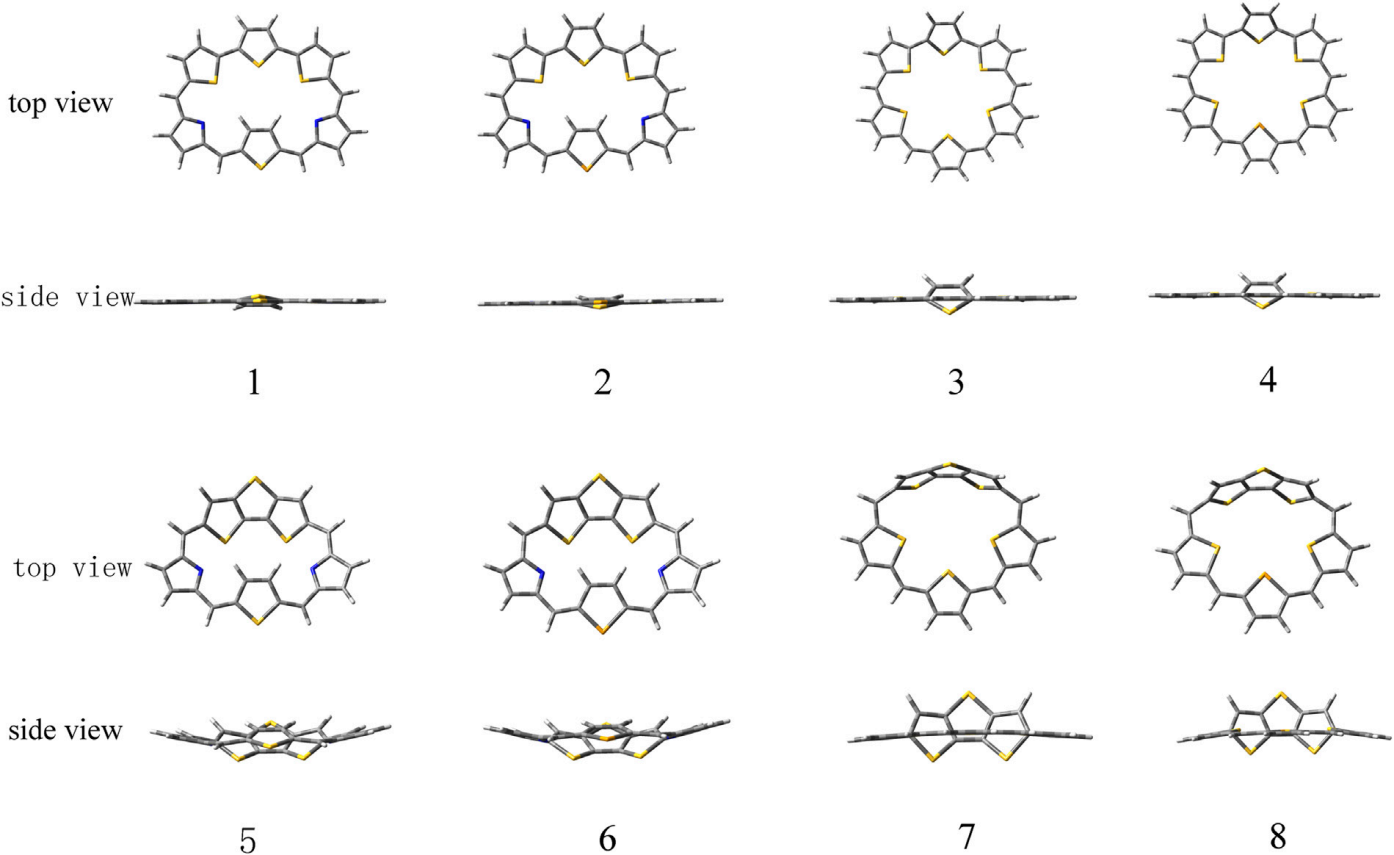
Optimized ground state structures of molecules **1–8**.

The ground state structures of the designed molecules are optimized at the B3LYP/6-311G (d, p) level of theory, as presented in Figure 1. The difference between TTH and DTT units lies in the presence of the C-C single-bond bridge in the former. The designed molecules are constructed by replacing the pyrrole fragment of the porphyrin ring with either a TTH unit (**1**–**4**) or a DTT unit (**5**–**8**). Molecules **1**, **2**, **5,** and **6** feature the N heteroatom in the pyrrole group, whereas molecules **3**, **4**, **7** and **8** contain the S heteroatom in the pyrrole group. The key geometrical parameters of all molecules are listed in Table 1.

**TABLE 1:** Main optimized geometry structure parameters of **1**–**8**.

	**1**	**2**	**3**	**4**	**5**	**6**	**7**	**8**
Bond lengths (Å)								
N7-N8	7.508	7.564			6.106	6.172		
S7-S8			6.520	6.578			5.175	5.394
Bond angles (°)								
C4-C5-C6	125.5	125.6	129.2	129.7	119.8	120.8	123.2	123.4
C9-C10-C11	128.5	129.3	133.1	133.5	122.6	123.4	129.8	130.6
Dihedral angles (°)								
S1-C4-C5-C6	0.0	0.2	1.2	1.9	20.6	21.2	65.3	71.5
N7-C9-C10-C11	0.0	0.0			16.1	17.1		
S7-C9-C10-C11			0.0	0.0			1.5	3.6

**SCHEME 2:**
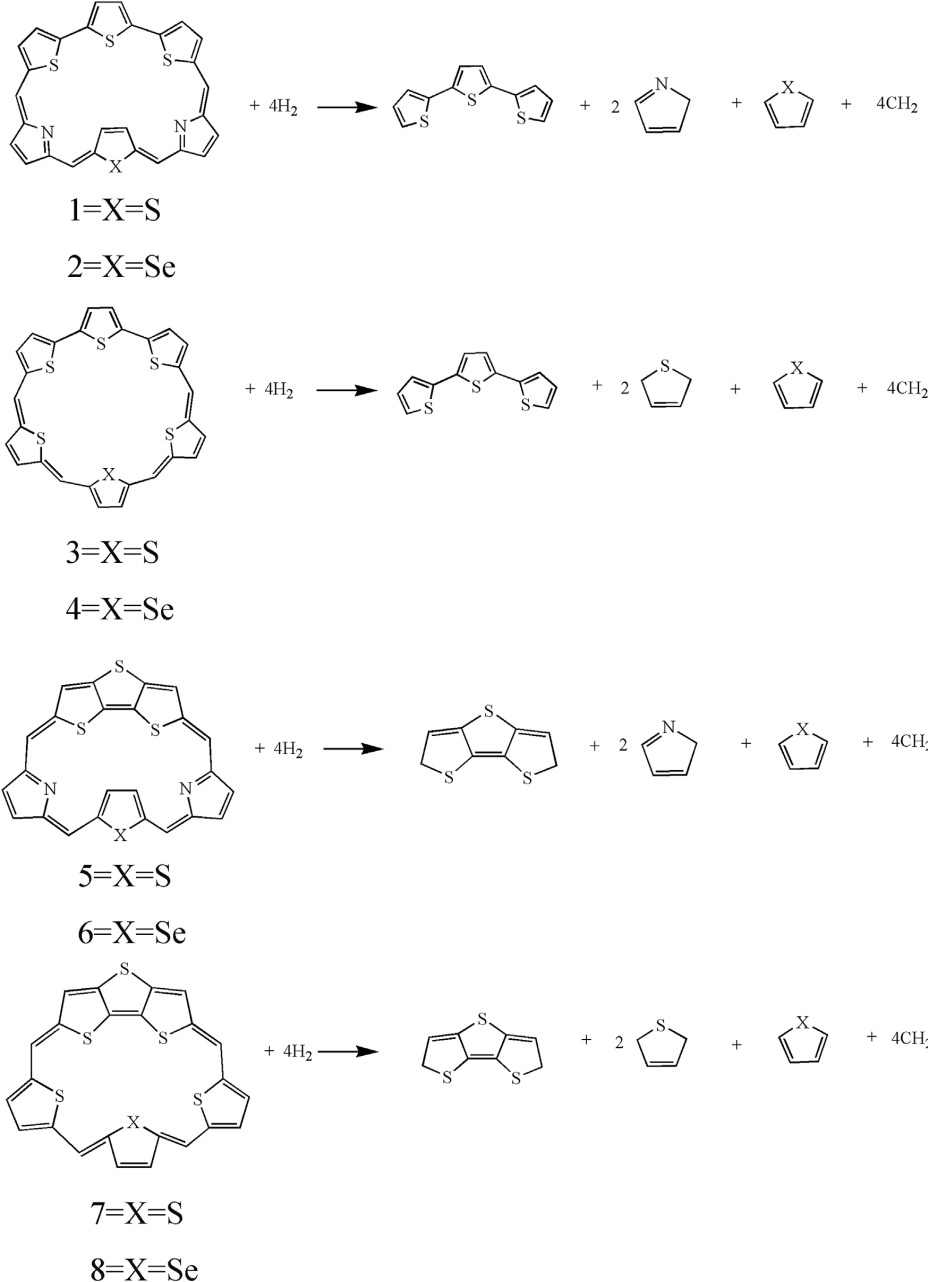
The isodesmic reactions of **1**–**8**.

It can be seen that the N_7_-N_8_ bond lengths of molecules **1**, **2**, **5,** and **6** are 7.058, 7.564, 6.106, and 6.172 Å, respectively. The S_7_-S_8_ bond lengths of molecules **3**, **4**, **7,** and **8** are 6.520, 6.578, 5.175, and 5.394 Å, respectively. It is obviously that both bond lengths increase in the order of **7** < **8** < **5** < **6** < **3** < **4** < **1** < **2**. For bond angles, C_4_-C_5_-C_6_ of molecules **1**-**8** is 125.5°, 125.6°, 129.2°, 129.7°, 119.8°, 120.8°, 123.2°, and 123.4°, respectively. The C_9_-C_10_-C_11_ bond angles of all molecules change following the same order as the C_4_-C_5_-C_6_ bond angles. The dihedral angles of S_1_-C_4_-C_5_-C_6_, N_7_-C_9_-C_10_-C_11_, and S_7_-C_9_-C_10_-C_11_ are listed in Table 1 as well. The dihedral angles of **1**–**4** are nearly zero, indicating that the four molecules would have better coplanarity. Figure 1 shows that **1**–**4** has planner structure, whereas **5** and **6** exhibit bowl structure. For **7** and **8**, the DTT unit is perpendicular to the porphyrin core, whereas the three thiophene units of the porphyrin core are nearly coplanar, as evidenced by the values of dihedral angles. The calculated ASE values are listed in Table 2. We choose TTH, DTT, and 5-membered rings as the reference structures that present localized single and double bonds (Scheme 2). Generally, the molecule with a larger (more negative) ASE value would have stronger stability. It is shown from Table 2 that the calculated ASE values decrease in the order of **2** > **1** > **4** > **3** > **6** > **5** > **8** > **7**, indicating that stability of molecules decreases in the same order.

**TABLE 2:** Computed aromatic stabilization energy (ASE, in kcal/mol) of molecules **1**–**8**.

	1	2	3	4	5	6	7	8
ASE	422.7	426.4	412.5	416.3	391.2	393.4	368.4	376.6

To gain a deep understanding of the stability, the aromaticity of atomic rings of all molecules is calculated. Positive values of NICS indicate antiaromaticity. The more negative (smaller) NICS value suggests better stability. The critical points are analyzed at the geometrical center of a ring or above a molecular plane. To avoid the in-plane components, the NICS values are calculated at 1 Å above the thiophene and pyrrole rings (points a, b, c, d, e, and f), molecular center (point k), and intramolecular (points g, h, i, and j) using the flow around the gauge-independent atomic orbital method. An illustration of the different critical points is detailed in Scheme 1. This approach has been widely used to evaluate the aromaticity and stability of porphyrins, including our and others’ works (Hong and Kim, 2003; Jeon et al., 2004).

The calculated NICS (1) values, as listed in Table 2, show that NICS (1) at 1 Å above the critical points a-k are all negative for molecules **1**–**8**. The NICS (1) values at 1 Å above the critical points a, b, and c for **1**–**4,** and critical points a and c for **5**–**8** are close. An exception is the NICS (1) value of **5**–**8** at 1 Å above the critical point of ring b show much smaller absolute values. It shows that the middle thiophene moiety in TTH has larger aromaticity than in DTT. In addition, the NICS (1) values of **1**–**8** at 1 Å above the critical points of rings d and f are close. The intramolecular 1 Å above critical points g, h, i, and j for **1**–**8** are similar. Moreover, comparisons of the NICS (1) values at critical point e for molecules with pyrrole N replaced by Se and S suggest that the inverted thiophene ring and selenophene ring are more stable than the conventional structure. As for the molecular center 1 Å above critical point, the NICS (1) values are −20.56, −21.21, −17.43, −18.12, −16.54, −16.91, −15.38, and −15.67 ppm for **1**–**8**, respectively, Table 3. It can be concluded from the aforementioned discussions that the aromaticity of all molecules increase in the order of **7** < **8** < **5** < **6**< **3** < **4**< **1** < **2**. We note that the NICS (1) value of porphyrin is only -14.98 ppm (Wei et al., 2012). This indicates that all designed molecules have strong aromaticity than porphyrin.

**TABLE 3:** NICS (1) values for **1**–**8**.

NICS (ppm)	**1**	**2**	**3**	**4**
a Thiophene ring NICS (1)	−19.68	−19.88	−22.57	−22.54
b Thiophene ring NICS (1)	−24.61	−20.83	−22.43	−21.04
c Thiophene ring NICS (1)	−22.89	−19.42	−21.41	−21.01
d Pyrrole/thiophene ring NICS (1)	−1.02	−1.35	−1.92	−1.81
e Thiophene/selenophene ring NICS (1)	−16.69	−18.62	−8.10	−8.93
f Pyrrole/thiophene ring NICS (1)	−1.47	−1.78	−1.18	−1.42
g Intramolecular critical point NICS (1)	−18.09	−17.20	−18.52	−19.74
h Intramolecular critical point NICS (1)	−17.28	−16.63	−18.74	−18.38
i Intramolecular critical point NICS (1)	−18.86	−16.87	−17.01	−19.92
j Intramolecular critical point NICS (1)	−18.00	−16.73	−17.41	−18.28
k Molecular ring left NICS (1)	−20.56	−21.21	−17.43	−18.12
**NICS (ppm)**	**5**	**6**	**7**	**8**
a Pyrrole ring NICS (1)	−21.95	−20.96	−18.35	−21.36
b Thiophene ring NICS (1)	−0.63	−0.29	−0.22	−0.56
c Thiophene ring NICS (1)	−19.95	−19.20	−18.18	−22.42
d Pyrrole/thiophene ring NICS (1)	−1.15	−0.68	−0.77	−0.92
e Thiophene/selenophene ring NICS (1)	−15.18	−15.85	−7.53	−7.96
f Pyrrole/thiophene ring NICS (1)	−1.13	−0.79	−0.68	−0.86
g Intramolecular critical point NICS (1)	−16.44	−17.16	−16.22	−17.02
h Intramolecular critical point NICS (1)	−16.20	−17.38	−16.83	−18.34
i Intramolecular critical point NICS (1)	−16.34	−17.74	−16.67	−19.02
j Intramolecular critical point NICS (1)	−16.40	−17.77	−16.07	−17.34
k Molecular ring left NICS (1)	−16.54	−16.91	−15.38	−15.67

**FIGURE 2:**
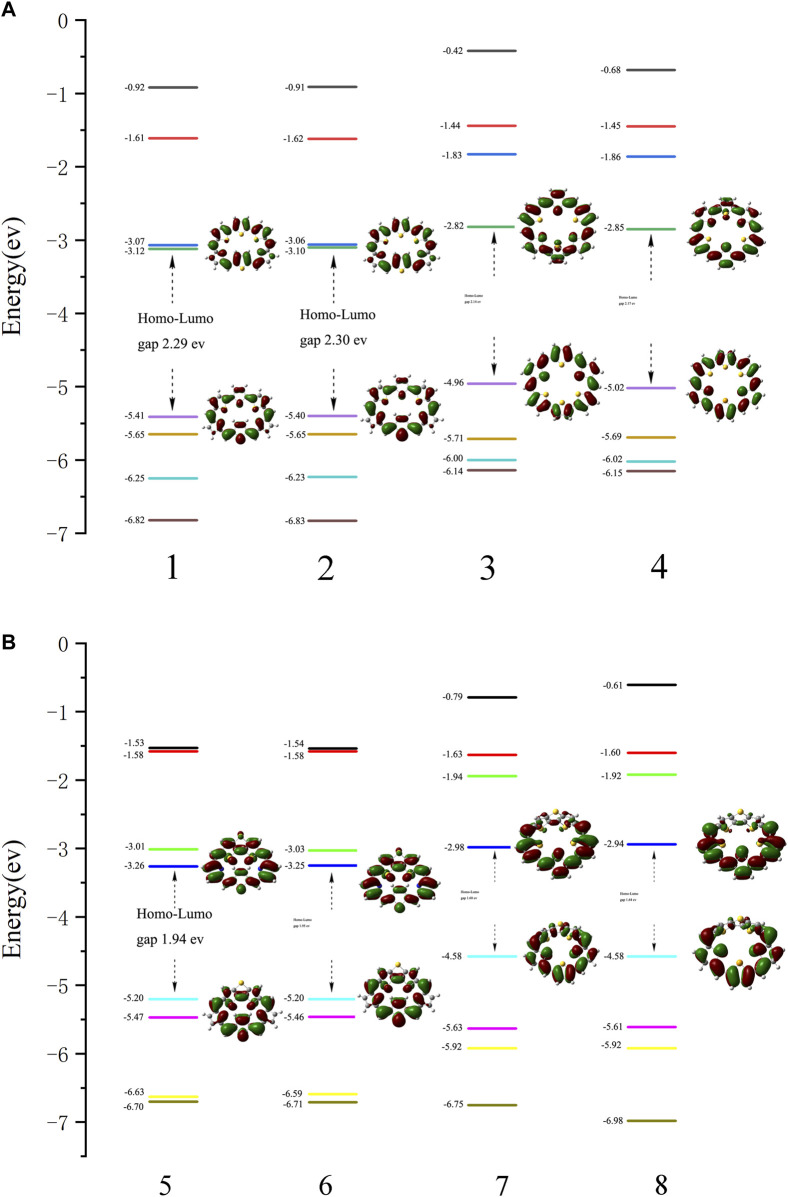
Molecular orbital distributions and diagrams of the frontier molecular orbitals (from HOMO-3 to LUMO+3) for **(A) 1**, **2**, **3,** and **4** and **(B) 5**, **6**, **7,** and **8**.

Energy levels of HOMO, LUMO, and their gap, Δ_H-L_, are also used to evaluate the stability of compounds. The Frontier molecular orbital diagrams and the Δ_H-L_ of molecules **1–8** are shown in Figure 2. Both the HOMOs and LUMOs spread over the whole π-conjugated backbones. For **1** and **2**, HOMO orbitals exhibit bonding characters, whereas LUMOs exhibit antibonding characters. In contrast to **1** and **2**, the HOMO of **3** and **4** show antibonding character, while the LUMO exhibits bonding character. For **5** and **6**, the HOMOs display antibonding characters in DTT, while the LUMOs display bonding characters in DTT. For **7** and **8**, the HOMOs display antibonding character at the bottom thiophene ring, while the LUMOs display bonding character at the bottom thiophene ring. A larger HOMO-LUMO gap indicates a more stable structure. The HOMO-LUMO gaps of **1** (2.29 eV), **2** (2.30 eV), **3** (2.14 eV), **4** (2.17 eV), **5** (1.94 eV), **6** (1.95 eV), **7**(1.60 eV) and **8** (1.64 eV) are listed in Figure 2. For **1–8**, molecules **1–4** have larger HOMO-LUMO gaps than **5–8**. A positive correlation between NICS (1) (in absolute value) and HOMO-LUMO energy gaps can be observed, that is the larger HOMO-LUMO gaps would have the more negative NICS (1) value. A similar observation has been reported in our previous works [33-34].

**FIGURE 3:**
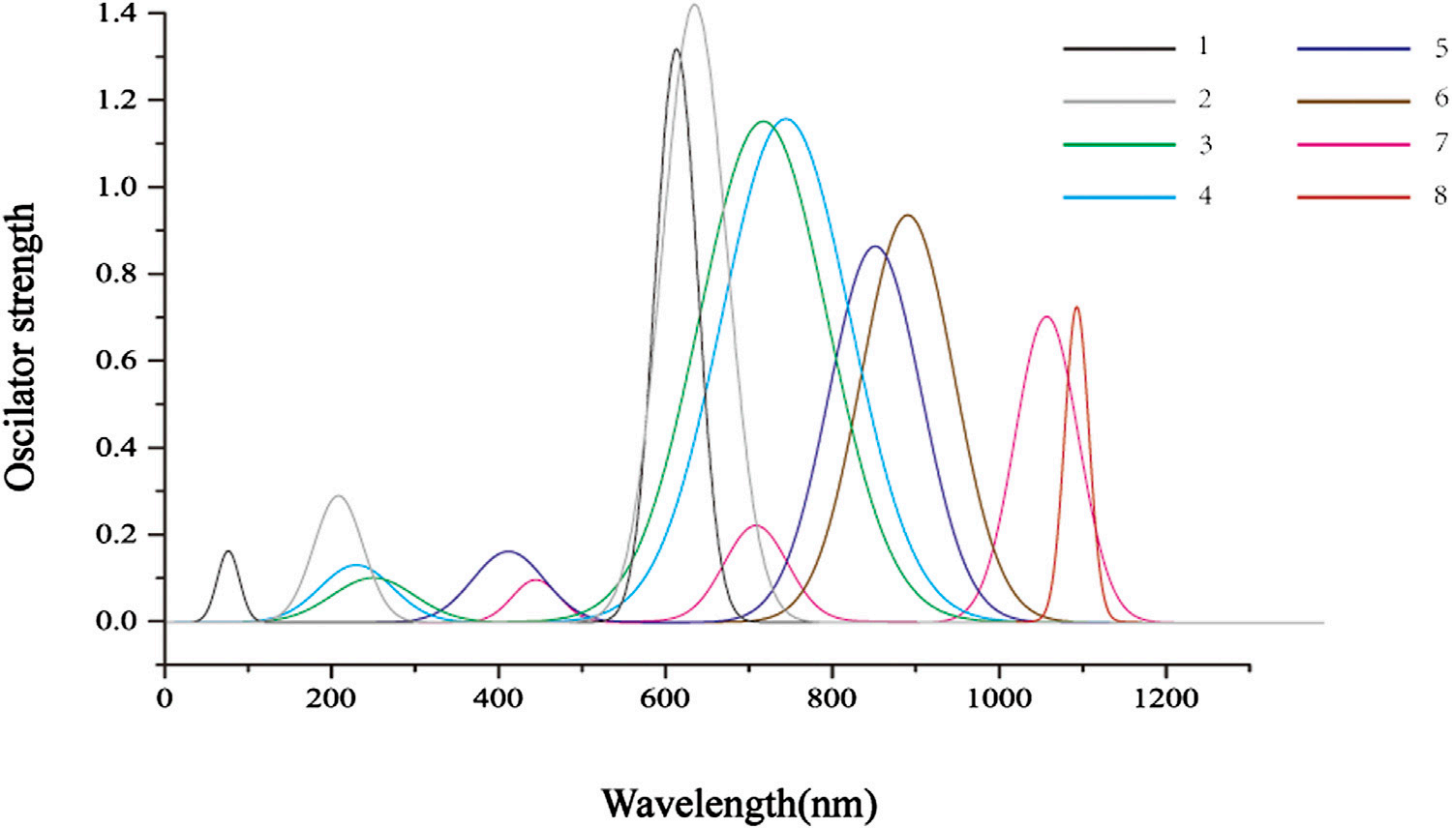
The simulated absorption spectra of molecules **1–8** were obtained using TD-DFT/CAM-B3LYP/6-311G (d,p) level of theory.

The TD-DFT method is used to calculate the vertical electronic excitations, as reported in Table 4. The simulated absorption spectrum is shown below. From Figure 3, it can be seen that, for all molecules, the major absorption bands are located in the visible light region. The dominated transitions for molecules **1** and **2** can be attributed to HOMO→LUMO + 1. For **3–8**, the major transitions are assigned to HOMO→LUMO. It is clear that the absorptions are significantly redshifted and intensified with the substitution of the pyrrole N atom by Se atoms as compared to one substitution by S atoms. One can also observe that the absorptions are significantly redshifted and broadened when incorporating DTT moiety. For **2**, the lowest-lying excitation is calculated at 707 nm, and the major transition of HOMO→LUMO has a maximum oscillator strength of 1.403.

**TABLE 4:** Calculated absorption features of **1**–**8**.

Molecules	E/nm (eV)	Major contribution	Oscillator strength
**1**	660 (1.88)	HOMO-1→LUMO (49%)	1.310
		HOMO→LUMO+1 (51%)	
**2**	707 (1.75)	HOMO-1→LUMO (48%)	1.403
		HOMO→LUMO +1 (51%)	
**3**	743 (1.67)	HOMO→LUMO (70%)	1.137
**4**	767 (1.62)	HOMO→LUMO (70%)	1.182
**5**	864 (1.44)	HOMO-1→LUMO+1 (39%)	0.896
		HOMO→LUMO (59%)	
**6**	896 (1.38)	HOMO-1→LUMO+1 (39%)	0.928
		HOMO→LUMO (59%)	
**7**	1049 (1.18)	HOMO-1→LUMO (17%)	0.695
		HOMO→LUMO (68%)	
**8**	1076 (1.15)	HOMO-1→LUMO (20%)	0.714
		HOMO→LUMO (67%)	

## Conclusion

We have investigated the aromaticity, stability, and photophysical properties of **1–8** with TTH and DTT units incorporated in the porphyrins using DFT and TD-DFT calculations. The stability of molecules is directly related to aromaticity, which can be evaluated using the Δ_H-L_ values and the NICS (1) values. The calculation results show that all the designed molecules show larger aromaticity and excellent photophysical properties. It is also revealed that the molecule with TTH unit is more stable than the one with the DTT unit, which can be ascribed to the coplanarity of the TTH unit. For the designed molecules, the one with an inverted thiophene ring or selenophene ring is more stable than the one with a thiophene ring or selenophene ring. Substitution of the pyrrole N atom by Se heteroatom results in a more stable structure than by S heteroatoms. All the absorption bands of the molecules are located in the visible region. The major absorptions are from either HOMO→LUMO + 1 or HOMO→LUMO transitions, which are beneficial for the intramolecular charge transfer process. Compared to the other molecules, compound **2** stands out because it shows unique properties, that is, larger HOMO-LUMO gap, better coplanarity, and stronger aromaticity. We expect these theoretical studies could pave the way for the future development of novel porphyrin molecules.

## Data Availability

All datasets generated for this study are included in the article/supplementary material.
